# Using an Intersectional Lens on Vulnerability and Resilience in Minority and/or Marginalized Groups During the COVID-19 Pandemic: A Narrative Review

**DOI:** 10.3389/fpsyg.2022.894103

**Published:** 2022-05-18

**Authors:** Heidi Siller, Nilüfer Aydin

**Affiliations:** Department for Psychology, University of Klagenfurt, Klagenfurt, Austria

**Keywords:** COVID-19 pandemic, social inequality, resilience, vulnerability, intersectionality, narrative review

## Abstract

Throughout the pandemic, the media and scholars have widely discussed increasing social inequality and thereby publicly pointed to often hidden and neglected forms of inequality. However, the “newly” arisen awareness has not yet been put into action to reduce this inequality. Dealing with social inequality implies exploring and confronting social privileges, which are often seen as the other side of inequality. These social constructs, inequality and privilege, are often discussed in light of vulnerability and resilience. This is particularly important in the context of the worldwide coronavirus disease 2019 (COVID-19) pandemic and efforts to end the pandemic, as both constructs are discussed regarding access to healthcare, vaccination, and education and knowledge, misinformation, social resources, economic resources, and so forth. Minority and/or marginalized groups may be particularly vulnerable to the impact of the COVID-19 pandemic. However, resilience factors in these groups may be neglected and underreported. This narrative review aims at illustrating the specific and intertwined aspects of resilience and vulnerability in minority and/or marginalized groups during the COVID-19 pandemic. To achieve this, we use an intersectional lens based on recommendations made by Moradi and Grzanka. A total of 48 articles were included in the narrative review. Most of them were commentaries focusing on social inequality, vulnerability, and/or resilience. Based on the dissection of articles at structural, systemic, and individual levels, we propose three hypothesis on vulnerability and resilience in minority and marginalized individuals and groups: (1) social inequality must be considered at a global level; inequality at a global level translates into a vulnerable context for an individual; (2) vulnerability is historically situated: vulnerability (experienced during the pandemic) is maintained and reinforced by history; (3) strength through collective (historical) hardship: vulnerability is not the opposite of resilience but may serve as an aspect of resilience. The conclusions drawn from this review show that we need to include diverse voices to advance concepts, such as vulnerability and resilience, in minority and marginalized groups. Additionally, these concepts are not necessarily in opposition to each other, but vulnerability should be understood as an integral part of resilience.

## Introduction

In March 2020, the coronavirus known as COVID-19 was declared a pandemic by World Health Organization [WHO] (2020a,b). In the opening remarks, the WHO director general was concerned by the lack of resources in some countries as well as the economic and social consequences that would result from the pandemic (World Health Organization [WHO], 2020b), thus indicating that social inequality, in terms of unequal distribution of or access to resources or positions and in terms of status or power, might be amplified by the pandemic. During the course of the pandemic, the social divide in societies has deepened, and social inequality has become more exposed (Kawachi, 2020). Thus, in this review, we focus on social inequality during the COVID-19 pandemic and explore the concepts of vulnerability and resilience in minority and marginalized groups.

Even before the pandemic, it was stated that “[t]he extent of inequality around the world is enormous” (p. 250) (Blackburn, 2008). Despite recognizing social inequality and its increase as problematic, it also “has become fashionable to ignore it” (p.250, ibid). The neglect of social inequality can be illustrated and comprehended with the Coin Model (Nixon, 2019). In this model, societal structures and systems of oppression, such as racism, classism, sexism, and ableism, are presented by a coin providing advantage or disadvantage for an individual depending on her/his group membership. Disadvantage for one social group usually means (unearned) privilege for another social group (Nixon, 2019). Advantages due to privilege are often considered to be based on meritocracy, thus cloaking privilege and inequality. However, again, the invisibility of privilege strengthens privilege in its power and persistence (Phillips and Lowery, 2020). Consequently, social inequality is not solely referring to an individual level but should be understood as the interplay between structural, systemic, and individual levels. In the sense of intersectionality, these levels are interconnected and intersecting. At the individual level, the social characteristics of diverseness, such as gender, ethnicity, and class, may intersect and create or shape inequalities resulting from social structures or systems of oppression (Crenshaw, 1989, 1991). At the structural and systemic levels, systems of oppression impact society and individuals. These systems relate to racism, sexism, heteronormativity, classism, ableism, homophobia, and so forth. The systems are also subject to structural forces from politics, history, legislation, economy, and colonialization, to name a few (McCollum et al., 2019). In this sense, we have to consider social characteristics in their interwovenness, in their relation to structures and systems, and as unique individual experiences. With such an understanding, we may gain insight into oppression and power and their expression in the form of privilege and inequality (Crenshaw, 1989; Warner and Shields, 2013; Hankivsky, 2014; Collins, 2015; Moradi and Grzanka, 2017).

Social inequality is often discussed with reference to minority and marginalized groups. These concepts are connected, as marginalized groups overlap with minority groups. Minority and majority groups are often defined in terms of, e.g., social categories, power, or group size (Seyranian et al., 2008). Marginalization, as defined by Hall et al. (1994), initially referred to individuals or social groups on the margins due to their identity or social characteristics, environment, associations connected to a social group, and experiences. Marginalization is thereby a process that limits access to and participation in power, social, and political roles. Since Hall et al.’s initial definition of marginalization, the concept has been expanded and adjusted to include intersecting identities and social characteristics, power relations, exclusion from dominant discourses, and globalization (Hall and Carlson, 2016). Marginalization is based on structural (e.g., laws), systemic (e.g., oppression, such as racism), and individual levels (e.g., discrimination) and the interaction between these levels (Baah et al., 2019).

On this note, it is apprehensible that experiencing or living in the pandemic is not the same for every one (Kawachi, 2020; Gubrium and Gubrium, 2021), but it is impacted by multiple individual and structural levels shaping everyday lives. The COVID-19 pandemic is often referred to as a crisis, a trauma (Bridgland et al., 2021), or a disaster [cf (Wisner et al., 2004)], which affects the individual, systemic, and structural levels of a society and which has national and global impacts. A crisis is a comprehensive concept, which includes trauma and disasters (Shaluf et al., 2003) and should thus be understood as a continuum (Dulmus and Hilarski, 2003). A crisis (e.g., critical turning points in the lives of individuals) is marked by its impact on individuals and, beyond the individuals, by its potential of being perceived as a threat and by disrupting life spaces (Eastham et al., 1970). A crisis may have negative and positive effects, but some concepts related to the crisis, e.g., traumatic stressors or disasters, are focusing mostly on negative aspects. It is not solely the event itself that is characterized as traumatic or stressful but also the perception of an event as stressful or traumatic (Dulmus and Hilarski, 2003), which might vary across individuals. Feminist views on trauma theory emphasized social locations and intersections in the construction of trauma (Burstow, 2003; Quiros and Berger, 2015) and multiple interpretations of the term “trauma” (Tseris, 2013). Black, postcolonial, and indigenous analyses added a social and political understanding of trauma to a clinical one, with the latter focused on (emotional) response to traumatic events (Pain, 2020, 2021). Social and political aspects can be located in collective trauma as a shared, collective experience and a transgenerational understanding of trauma (Pain, 2020), including the impact of trauma due to membership to specific groups (Burstow, 2003). In this sense, trauma has to be considered in the context of oppression, in which oppression is a traumatizing component (Burstow, 2003). This view is particularly meaningful for minority or marginalized groups.

In the context of the pandemic, social inequality coincides with vulnerability to the pandemic and vulnerability due to the impact of the pandemic. Vulnerability can be defined as “characteristics of a person or a group and their situation that influence their capacity to anticipate, cope with, resist, and recover from the impact of a natural hazard” (p. 11) (Wisner et al., 2004). Combinations and intersections of social characteristics, social systems, and structural elements shape risk and vulnerability to hazards (e.g., the pandemic). They impact the access to resources and (unequal) exposure to hazards (Wisner et al., 2004). This underlines that experiencing the pandemic and its impact is, among other aspects, influenced by social inequality.

Vulnerability is usually seen as something undesirable and has the notion of resulting in “a barrier between two social worlds, which isolates and marginalizes the wounded.”(p. 255) (Baiasu, 2020). Baiasu (2020) highlights that such an understanding of vulnerability also creates asymmetry, stigmatization, and marginalization. Vulnerability is also seen as pre-event aspect, which affects the chance of experiencing risk or harm (Cutter et al., 2008). Subsequently, the concept of resilience may be seen as a promise to overcome vulnerability and stigmatization, as resilience is often considered as a counterpart of vulnerability. In the context of mental health and social sciences, resilience has received considerable attention since its conceptualization. In the 1970s, resilience was observed in children growing up in adverse environments. Emmy Werner et al. found that not all of these children entered a vicious circle of adversity, violence, or crime, but some of them grew up to be mentally healthy and “successful” adults (Werner and Smith, 1982, 1992; Werner, 1989). Since then, resilience has been conceptualized as, e.g., individual, psychological, social, ecological, and community resilience (Quinlan et al., 2016), each highlighting specific aspects of resilience. In particular, a review on social resilience in the context of disaster concluded that resilience refers to the ability of social entities (e.g., individuals, families, organizations, and communities) that are connected to social mechanisms to cope, withstand, and/or recover from disasters (Saja et al., 2021). Such an understanding of resilience shows the interconnection to vulnerability. In the context of vulnerability social characteristics and mechanisms impede the capacity to cope, withstand, or recover (Wisner et al., 2004), whereas resilience refers to the interplay of mechanisms and characteristics enabling this very capacity (Saja et al., 2021). In this viewpoint, the social system “absorbs” the impact of a hazard (Cutter et al., 2008). Additionally, the adaptive function of resilience affects the time after a disaster. Nevertheless, resilience is not a stable and fixed phenomenon but is dynamic in nature and might vary over time (Cutter et al., 2008; Saja et al., 2021). Moreover, resilience is a contested concept (Davoudi et al., 2012), critiqued for centering on ableism, hegemony, and positivism (Hutcheon and Lashewicz, 2014). For example, resilience is embedded in a socially constructed context of crisis (Davoudi et al., 2012) and, thus, constructed itself. In this sense, resilience should be understood in its context with regard to subjectivity, meaning-making, and its potential to resume or increase connectivity (Hutcheon and Lashewicz, 2014). A socio-constructivist understanding of resilience allows for understanding the concept with its diverse trajectories and shapes. This perspective extends the concept beyond its (critiqued) normative function.

In this review, we focus on the understanding of resilience as an interplay between social systems and individuals. We see resilience as a capacity to adapt in times of adversity and as embedded in social processes that enable the process of resilience (Juen and Siller, 2013). Consequently, the resilience of an individual is understood in the context of social systems (e.g., community and state), which provide resources for the individual. We acknowledge that resilience is shared but also subjective and constructed. Multiple understanding of resilience and co-existing narratives of resilience illustrate the diversity and socio-constructivist nature of resilience as a concept (Powell et al., 2014). As can be derived from the definitions of vulnerability and resilience, vulnerability is ascribed to the context in which individuals and groups live, often labeled as disadvantaged, minority or marginalized, pre-event, whereas resilience is referred to during or post-event. In the context of vulnerability, we acknowledge that not individuals or groups are vulnerable, but that the situation and structures in which they are embedded create vulnerability.

As drafted in this introduction, we seek to understand vulnerability and resilience during the COVID-19 pandemic using an intersectional lens. By means of a narrative literature review, we explore these concepts in minority and marginalized groups in the context of social inequality.

## Methods

### Using an Intersectional Lens

Intersectionality is sometimes discussed as a method (Bowleg, 2008; Winker and Degele, 2011), tool (Mattsson, 2014), framework (Hankivsky et al., 2014), lens, or even theory (Cho et al., 2013). In this context, we will use intersectionality as a lens to view and perceive scholarly literature on social inequality during the pandemic. We thereby draw this intersectional lens on recommendations made by Moradi and Grzanka (2017). These recommendations include reflecting on epistemological aspects, thus reflecting self-evident views on knowledge production and procedures, working interdisciplinary to challenge epistemological assumptions and biases, not limiting inequality to specific social groups and assuming majority or dominant social group as the reference group, and considering structures and mechanisms, as well as systems and dynamics in inequality over-focusing on individual aspects and identities (Moradi and Grzanka, 2017). The latter recommendation is particularly relevant in psychology (our institutional embedding), which, as a discipline, is often focused on individuals and groups but less on societal structures and social systems in which inequality is embedded.

In more specific terms, we first searched the literature on social inequality and vulnerability or resilience. After screening in several circular steps to narrow down the most relevant, the literature was dissected with an intersectional lens. We focused on concepts or social groups in the context of social inequality, discussion of structures or mechanisms producing and maintaining inequality, and the way vulnerability or resilience was produced or reproduced. During this process, our own reflections on our positionality and epistemology were used as stimulants to enrich the analysis and discussion of the literature.

### Reflexivity

Discussing social inequality equates to listening to marginalized and, often, silenced voices. The intentional or unintentional replication of oppression is to be avoided when researching and discussing social inequality. It is preferable to have marginalized voices shaping research, and it is necessary to reflect on one’s self-conception in relation to the field of research. Particularly in the sense of intersectionality and from a feminist point of view, this endeavor includes uncovering own standpoints in research and in the subject of research. Feminist standpoint theory demands us to reflect and acknowledge our standpoints from which we speak about inequality (Harding, 1987, 1992; Haraway, 1988). Such reflexivity is understood as an integral part of the research, which should be explicit and go beyond mere lip service of stating one’s social location (Sweet, 2020).

In this sense, the first author (HS) is a female and White researcher in a predominantly White academic environment. Her identity as cis-gendered, west-European socialized, middle-class woman provided her with limited experience of inequality. Experiencing inequality included sexism and an often encountered male preference in the academe. Her interest in social inequality and intersectionality arose from a gender inequality and heteronormative standpoint. Additionally, her research is informed by being trained in psychology but most often by working in interdisciplinary context, such as medicine, sociology, or pedagogy. This also informs the current dissection and sense-making of the scholarly literature in this review.

The second author (NA) is a sociologist working in (social) psychology and is interested in how different forms and mechanisms of social exclusion (e.g., interpersonal rejection, stigmatization, or discrimination) impact an individual at a group and a societal level. Exclusionary mechanisms have powerful negative consequences on individuals’ physical and mental health. They shape behavioral responses like interpersonal aggression, antisocial behavior, and even (political) radicalization, resulting in reduced opportunities for successful societal participation. In contrast, social inclusion and connectedness foster social justice by contributing to individual well-being, cooperation, and prevention of social deprivation. Thus, the author is motivated by a social justice agenda that, in general, encourages research on this feminist standpoint.

## Search Strategy

To include relevant articles, several databases were searched. These databases were Web of Science and Core Collection, and *via* Ovid^®^ we searched APA PsychArticles, APA PsychInfo, and Ovid Medline(r).

Search terms included “social inequality” or “privilege,” “minor*” or “marginal*,” and “COVID-19” or “corona.” On Web of Science, this search strategy yielded 45 results. Of these, 1 was double, 4 were not related to COVID-19, 2 were in Spanish, and 1 could not be retrieved. *Via* Ovid^®^, 66 results were found. Of these, 4 were removed because they were duplicates, 24 were not related to COVID-19, and 9 did not focus on social inequality.

An additional search on the Web of Science included resilience and inequality (all fields) and COVID-19 or corona (all fields) and marginal* or minor* (all fields) and resulted in 12 results, *via* Ovid^®^, this search strategy yielded 28 results. Of the 28 results, 16 were not related to COVID-19 or inequality, and 1 was a proceeding. To double-check, another search was performed on resilience (all fields) and COVID-19 or corona or pandemic (all fields) and social inequality or minor* or marginal* (all fields). This search strategy yielded 205 results, 58 of these were included after initial screening of the titles. Despite not limiting the search to any specific language, only English articles were obtained. The inclusion of English articles only contributes to silencing other voices in this area. Unfortunately, discussion of such matter (e.g., implications of English as “lingua franca” in academic publications) is out of the scope of this article [see, e.g., (Pronskikh, 2018; Soler, 2019)].

Of the remaining 147 articles, the abstracts were read to determine inclusion in the narrative review. The inclusion criteria included focus on (1) vulnerability (explicitly) and/or resilience during the COVID-19 pandemic, (2) minority or marginalized social groups or individuals, and (3) social inequality and/or privilege. We searched for articles that discussed vulnerability and resilience.

**FIGURE 1:**
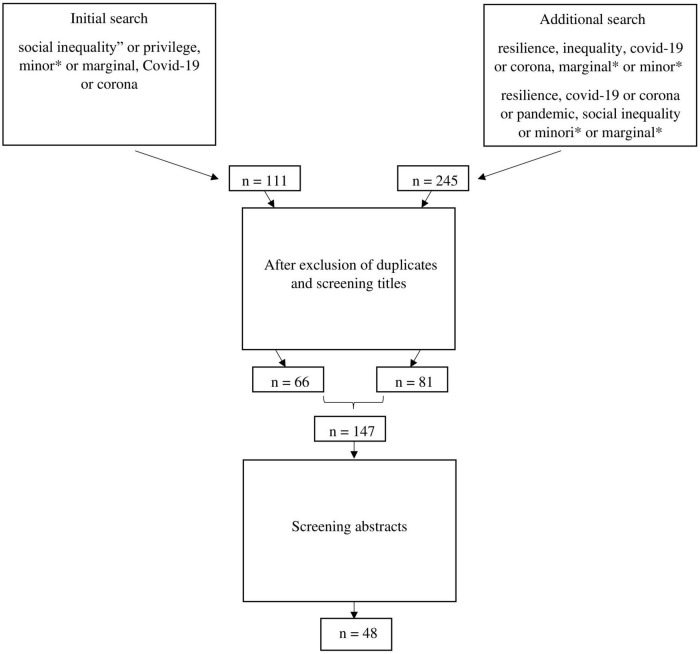
Search strategy.

The exclusion criteria included a general discussion of distress and vulnerability during the pandemic, no specific focus on vulnerability or/and resilience but on social justice, and increased social divide or increased inequality. Articles were excluded if they did not focus on social inequality, did not discuss social groups, or focused on something else than COVID-19 (also refer to Figure 1).

Overall, 48 articles were included in this narrative review. Most of the studies were conducted in the United States (n = 24), followed by the United Kingdom (n = 4), Israel (n = 4), and Canada (n = 4). The articles included surveys on several countries (n = 4), followed by reviews with no specific (named) country-specific domination (n = 2). Furthermore, articles focused on Malaysia (n = 2), China (n = 1), Germany (n = 1), Latin America (n = 1), and Nigeria (n = 1). Most of the articles focused on either exclusively or several of the following categories of social inequality: racism or ethnic/racial inequality (*n* = 19), LGBT, LGBTQI +, LGBTQI2S +, and sexual and gender minorities (*n* = 10), followed by diverse “vulnerable” groups (*n* = 5), socio-economic status, poverty, class (*n* = 9), ageism, older or elderly people (*n* = 2), chronic mental illness or psychiatric patients (*n* = 3), migrant workers, refugees, migrants (*n* = 2), chronic illness (*n* = 2), and sex workers (*n* = 1). Given the relatively short existence of the pandemic, 19 of the articles were of empirical nature, 26 were commentaries, perspectives, or (non-systematic) reviews, one was a systemic review, and one was a review on media and publications. An overview of the articles can be found in Table 1.

**TABLE 1:** Overview of included articles.

No.	Authors	Country	Category of social inequality	Vulnerability/ Resilience	Type of article
1.	Abreu et al., 2021	United States	LGBT, LGBTQI+, LGBTQI2S+, sexual and gender minorities	R	Empirical study (qualitative)
2.	Ahmed and Jackson, 2021	United States	Diverse vulnerable groups	V	Commentary, perspective, review
3.	Andia and Chorev, 2021	Latin America	Socio-economic status, poverty, class	V	Commentary, perspective
4.	Aung et al., 2021	Malaysia	Migrant workers, refugees, migrants	V	Empirical study (quantitative)
5.	Bhaskar et al., 2020	United Kingdom dominated	Racism, racial/ethnic inequality Socio-economic status, poverty, class	V	Commentary, perspective, review
6.	Bikomeye et al., 2021	United States	Racism, racial/ethnic inequality	V	Commentary, perspective, review
7.	Blustein et al., 2021	United States	Diverse vulnerable groups	V + R	Commentary, perspective, review
8.	Buffel et al., 2021	United Kingdom	Ageism, older or elderly people	V	Commentary, perspective, review
9.	Chackalackal et al., 2021	South Korea, Mexico, Colombia, India, Nigeria, and Nepal.	Diverse vulnerable groups	V	Literature review on media and publications
10.	Cheah et al., 2021	United States	Racism, racial/ethnic inequality	V	Empirical study (quantitative)
11.	Chen et al., 2020	United States	Racism, racial/ethnic inequality	V	Commentary, perspective, review
12.	Cheng et al., 2021	United States	Racism, racial/ethnic inequality	R	Commentary, perspective, review
13.	Cohen et al., 2020	Israel	Racism, racial/ethnic inequality	R	Empirical study (quantitative)
14.	D’Amico et al., 2020	United States	Racism, racial/ethnic inequality	R (+V)	Empirical study (quantitative and qualitative)
15.	Daud, 2021	Malaysia	Socio-economic status, poverty, class	V	Commentary, perspective, review
16.	Diaz et al., 2021	United States	Chronic mental illness, psychiatric patients	V	Commentary, perspective, review
17.	Frisina Doetter et al., 2021	United States	Racism, racial/ethnic inequality	V	Empirical study (quantitative)
18.	Gao and Sai, 2021	United Kingdom	Racism, racial/ethnic inequality	V	Commentary, perspective, review
19.	Garcia, 2021	United States	Racism, racial/ethnic inequality	V	Commentary, perspective, review
20.	Gibson et al., 2021	China and United States	Chronic mental illness, psychiatric patients	V	Systematic review
21.	Goldbach et al., 2021	United States	LGBT, LGBTQI+, LGBTQI2S+, sexual and gender minorities	V + R	Empirical study (quantitative)
22.	Gonzalez et al., 2021	United States	LGBT, LGBTQI+, LGBTQI2S+, sexual and gender minorities	R	Empirical study (qualitative)
23.	Haase, 2020	Germany	Socio-economic status, poverty, class	V	Commentary, perspective, review
24.	Herbers et al., 2021	United States	Socio-economic status, poverty, class	R	Commentary, perspective, review
25.	Hiebert and Kortes-Miller, 2021	Canada	LGBT, LGBTQI+, LGBTQI2S+, sexual and gender minorities	R	Empirical study (analysis of videos)
26.	Hunt et al., 2021	United States	LGBT, LGBTQI+, LGBTQI2S+, sexual and gender minorities	V + R	Empirical study (quantitative)
27.	Jones et al., 2021	United States	Racism, racial/ethnic inequality	V	Empirical study (qualitative)
28.	Kimhi et al., 2020	Israel	Racism, racial/ethnic inequality	R + V	Empirical study (quantitative)
29.	Kira et al., 2021	Arabic countries	Diverse vulnerable groups	V	Empirical study (quantitative)
30.	Krishnan et al., 2020	United States	Racism, racial/ethnic inequality	V + R	Commentary, perspective, review
31.	Lam, 2020	Canada	Sex workers	V (+R)	Commentary, perspective, review
32.	Leeming et al., 2022	United Kingdom	Chronic mental illness, psychiatric patients	V + R	Empirical study (qualitative)
33.	Lotta and Kuhlmann, 2021	Germany and Brazil	Migrant workers, refugees, migrants	V	Commentary, perspective, review
34.	Mahon and Mahon, 2021	general	Diverse vulnerable groups Racism, racial/ethnic inequality	R	Commentary, perspective, review
35.	McElroy-Heltzel et al., 2022	United States	Ageism, older or elderly people Socio-economic status, poverty, class Chronic illness	R (+V)	Empirical study (quantitative) (structured interviews)
36.	Mitchell et al., 2022	United States	LGBT, LGBTQI+, LGBTQI2S+, sexual and gender minorities	R (+V)	Empirical study (qualitative and quantitative)
37.	Morgan et al., 2022	Canada	Racism, racial/ethnic inequality Socio-economic status, poverty, class	R	Commentary, perspective, review
38.	Oginni et al., 2021	Nigeria	LGBT, LGBTQI+, LGBTQI2S+, sexual and gender minorities	V + R	Commentary, perspective, review
39	Poteat et al., 2020	United States	LGBT, LGBTQI+, LGBTQI2S+, sexual and gender minorities Chronic disease (HIV)	V (+R)	Commentary, perspective, review
40	Quinn et al., 2021	United States	LGBT, LGBTQI+, LGBTQI2S+, sexual and gender minorities	R	Empirical study (quantitative and qualitative)
41.	Saban et al., 2020	Israel	Racism, racial/ethnic inequality	R	Empirical study (quantitative analysis of registry data)
42.	Salerno et al., 2020	United States	LGBT, LGBTQI+, LGBTQI2S+, sexual and gender minorities	V	Commentary, perspective, review
43.	Sanchez et al., 2021	−	Socio-economic status, poverty, class	V	Empirical study (assessing jobs)
44.	Slobodin and Cohen, 2020	Israel	Racism, racial/ethnic inequality	V + R	Commentary, perspective, review
45.	Sullivan et al., 2021	United States	Racism, racial/ethnic inequality	V + R	Commentary, perspective, review
46.	Walubita et al., 2021	United States	Racism, racial/ethnic inequality	V	Commentary, perspective, review
47.	Waruszynski et al., 2021	Canada	Diverse vulnerable groups	V (+R)	Commentary, perspective, review
48.	Wu et al., 2021	China	Socio-economic status, poverty, class	R	Empirical study (quantitative)

*LGBT (lesbian, gay, bisexual, transgender), LGBTQI+ (lesbian, gay, bisexual, transgender, queer/questioning and others), LGBTQI2S+ (lesbian, gay, bisexual, transgender, queer/questioning, two-spirited and others); V (vulnerability), R (resilience). In row vulnerability and resilience V + R indicates equal focus on both concepts, if one is missing there was no focus on this concept and if one is put in brackets, the focus was less on this concept.*

## Findings

### Vulnerability

**FIGURE 2:**
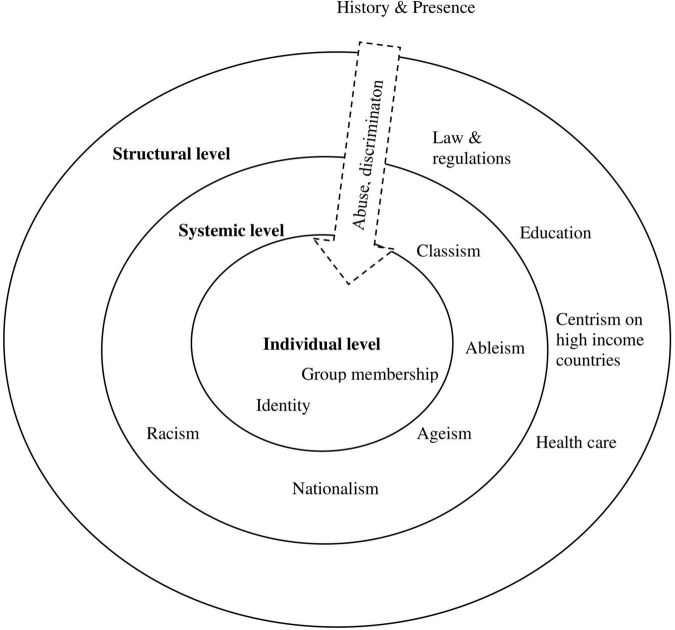
Impact on vulnerability across levels.

Vulnerability is used in terms of greater susceptibility to infection, adverse course of the disease, and mortality due to COVID-19 as well as vulnerability in terms of being adversely affected by measures established to contain the virus. Hence, vulnerability is influenced by different factors at the structural, systemic, and individual levels. These levels interact (refer to Figure 2) and increase or deepen social inequality. In the following first step, we discuss factors that intensify disparities. In the second step, we illustrate the concept of resilience in this context.

#### Structural and Systemic Levels: Government and State

Vulnerability and social inequality included the structural and systemic levels simultaneously. The intersection of racism, low income, and classism is considered in the discussion of (racial/ethnic) inequality during the COVID-19 pandemic. Particularly structural and systemic racism creates socioeconomic disadvantages in terms of lower income or housing locations as discussed in an article from the United States (Bikomeye et al., 2021). In Germany, Haase (2020) pointed out that discrimination-free access to green spaces is shaped differently across groups, thereby emphasizing socioeconomic status and poverty. In the United States, access to green spaces was more often seen on the nexus to racism, which was related to low socioeconomic status (Bikomeye et al., 2021; Garcia, 2021). In Latin America, inequality in resource allocation and the lack of financial aid by the state resulted in the higher vulnerability of the population (Andia and Chorev, 2021). In this context, the state had the ability to increase or mitigate the vulnerability. Similar conclusions were observed in Malaysia, where authors discussed that the successful reduction of poverty before the pandemic was reversed by the impact of the pandemic on the population. Also in this context, state-led social protection plans were seen as one basis to mitigate socioeconomic vulnerability (Daud, 2021).

#### Structural and Systemic Levels: Education, Healthcare, and Housing

The COVID-19 pandemic uncovered racial/ethnic inequality worldwide. Additionally, recent social movements, such as #Blacklivesmatter, accentuated racism in its systemic and structural forms and its impact on individuals as reflected upon in a United States article (Bikomeye et al., 2021). These seemingly parallel running crises, the Black lives matter movement and the COVID-19 pandemic, are interconnected, as both are deeply rooted in structural and systemic racism. Structural racism shapes the life and well-being of Black and Indigenous people and people of color (BIPOC) and reveals disadvantages in the lives of BIPOC. In this context, systemic racism is embedded in institutions of education, health, or legal regulations. Thus, racism, in its structural and systemic forms, impacts communities and individuals in profound and diverse ways. Authors of the reviewed literature highlighted the impact of racism on education and schools, health, and healthcare, as these areas are prominently affected by the pandemic and measures to contain the spreading of the virus.

The intersection of racism with socioeconomic status was linked to the impact mitigating strategies had on vulnerability to the virus. Racial/ethnic inequalities were anchored in historical and structural disadvantages for BIPOC individuals, which resulted in lower income as shown in the Canadian context (Morgan et al., 2022). COVID-19 measures targeted individual responsibility and individual behavior by focusing on physical distancing, staying at home, washing hands, or/and wearing masks. These measures did and do not acknowledge structural aspects impeding the possibility to follow these recommendations, e.g., in the German and United States context (Haase, 2020; Ahmed and Jackson, 2021). At structural levels, impediments included crowded housing, dense cities, and employment and working conditions across several countries, such as Germany, South Korea, Mexico, Colombia, India, Nigeria, Nepal, and Malaysia (Haase, 2020; Aung et al., 2021; Chackalackal et al., 2021). Crowded housing and dense cities create fewer possibilities for physical distancing, thus increasing infections with COVID-19; additionally, such crowded housing arrangements are often connected to lower-income, which created a greater divide between people with different socioeconomic statuses and increased social and socio-spatial inequalities. Urban green spaces as resources for recreation are not equally accessible for all social groups in many countries. This meant that urban green spaces usually contributing to well-being and quality of life were not accessible for marginalized groups if green spaces were closed for the public. Such green spaces are particularly important in times of crises, when recreational facilities (e.g., swimming pools and athletic facilities) were closed as discussed in German and United States articles (Haase, 2020; Bikomeye et al., 2021). Closures as mitigating measures potentially increased vulnerability in lower-income, crowded, and dense housing areas, as these living arrangements were not considered. Additionally, jobs that can be performed at home, are unequally distributed across countries and states (Sanchez et al., 2021). This demonstrates that systems and structures reproducing vulnerability and the maintenance of privilege and oppression have to be discussed at a global level (Sanchez et al., 2021).

In addition to current inequalities, United States perspectives emphasized that historic events influence vulnerabilities (Krishnan et al., 2020; Garcia, 2021) regardless of elapsed time. For example, United States-based commentaries pointed out that racism toward BIPOC patients deteriorates trust in the health care system (Walubita et al., 2021); recounts of the 1918 influenza pandemic reveal neglect and erasure of BIPOC in documentation of diseases and civic and healthcare participation. Consequently, disparities in health are sustained, emphasizing the need to collect diverse health data that go beyond the “norm” individual as critiqued in a United States article (Krishnan et al., 2020). As recited above, health disparities and vulnerability to diseases may be better inquired with a biological-social model (Garcia, 2021). This, in turn, acknowledges vulnerability beyond biological aspects but in interaction with intersections of structural, systemic, and individual relationships (Ahmed and Jackson, 2021). For example, based on the United States context, Garcia (2021) recounted the effect of the biological-social reinforcement or interaction on health, whereby biological aspects (such as chronic illness) interact or are exacerbated by social inequality (such as poverty).

#### Systemic Levels: Racism, Classism, and Schools

Concerns about racism in United States schools and education (D’Amico et al., 2020; Jones et al., 2021; Sullivan et al., 2021) focused on a dominant (hence White) group as norm when educating and teaching. Such approach to teaching was equivalent to invisibility of social resilience in diverse ethnic/racial groups (Mahon and Mahon, 2021), colorblindness (Jones et al., 2021), and lack of community cohesion or connection to traditional practices (D’Amico et al., 2020). This effect was even more pronounced in social groups with low socio-economic status as illustrated in the United States context (Herbers et al., 2021). COVID-19 measures, such as distance learning, deprived schools and education systems of additional benefits, such as nurturing relationships and routines (Herbers et al., 2021), interactions, sense of community, and a potential room for cultural connection and motivation for learning (Mahon and Mahon, 2021). This increased the potential vulnerability of some groups. However, schools and education cannot be treated as isolated entities. For example, Cheah et al. (2021) found in their study on 211 Chinese-American adolescents and parents in the United States that increased racial discrimination led to greater internalizing difficulties in the adolescents. However, this effect was mediated by biracial identity harmony and blendedness. Nevertheless, if parents warned against interacting with other racial-ethnic groups, adolescents also reported more internalizing difficulties. Racism in education is not limited to school settings and extends beyond this context. For example, in academia, anti-Asian racism was reported in a reflective piece from the United Kingdom (Gao and Sai, 2021), thereby demonstrating that racism also affects higher education across countries.

Schools and education as resource hubs can only function when actively including communities to counteract the effects of systems of oppression, such as racism. In this sense, different communities have different needs to survive and recover from the impact of the pandemic. To support diverse students, teachers should possess general awareness and acknowledgment of mechanisms of privilege and inequality. However, this does not appear to be the case. For example, a qualitative study with 42 school staff members in the United States showed that the staff members observed a connection between racial inequality and well-being and school achievements in their students but did not connect these to systemic or structural racism (Jones et al., 2021). Rather, they saw this effect resulting from low socioeconomic status. This highlights even more that there is a need to uncover the intersection of systemic mechanisms impacting individuals.

#### Systemic Level: Racism, Classism, and Health

Awareness of the impact of racism on health was focused upon in several publications. The observation related to different health systems and, in particular, systems in the United States was illustrated (Ahmed and Jackson, 2021). In general, the United States literature focused on the impact of structural and systemic racism, discrimination, and abuse on accessing and trusting healthcare (Chen et al., 2020; Ahmed and Jackson, 2021; Frisina Doetter et al., 2021; Garcia, 2021; Walubita et al., 2021). Healthcare systems are not neutral entities. Experiences of abuse, discrimination, or mistreatment, whether individual, collective, historical, or transgenerational experience, shape access to healthcare. In this context, access to healthcare is related with financial resources, healthcare availability, and trusting the medical and healthcare systems to care for a person. The impact of social inequality on COVID-19-associated vulnerability differs across ethnic/racial groups. It was shown that in Black and Hispanic communities in the United States, vulnerability due to social determinants and COVID-19 risk factors were significantly correlated with mortality in Black and Hispanic people but not in White people (Frisina Doetter et al., 2021). Additionally, several researchers from the United States, Canada, and Arabic countries have illustrated how racial discrimination worsened health (Chen et al., 2020; Kira et al., 2021; Waruszynski et al., 2021). Also, the social status within a social hierarchy in society contributed to vulnerability; in turn, the impact of the pandemic also influenced one’s status in the social hierarchy in, e.g., Arabic countries (Kira et al., 2021). In this sense, racism, classism, and health are interconnected in shaping vulnerability.

#### The Individual Level: Focus on Intersecting Identities

When it comes to various minority groups and individuals, intersections of identities and social characteristics are highlighted that may stimulate vulnerability and marginalization. For example, Arab minority communities in Israel showed lower infection rates, which was (hypothetically) connected to younger age, social media use (in comparison to ultra-Orthodox Jewish communities that more often deter from social media use), cooperation between community leaders and governmental bodies, and distribution of medical knowledge due to the higher percentage of medically trained people in the community (Saban et al., 2020). Other studies on the Arab community in Israel showed a picture consistent with effects seen in other minority groups: higher psychological distress, less resilience (Kimhi et al., 2020) but more confidence in the medical and healthcare systems, especially in suburban communities than in urban communities (Cohen et al., 2020). Overall, national identity struggles, discrimination, and social inequality in ethnic minority groups in Israel (Slobodin and Cohen, 2020) and Arabic countries (Kira et al., 2021) were exacerbated in the pandemic. These considerations bear resemblance to the discussion of racism in the United States.

Furthermore, minority and marginalized groups refer to older people, individuals with chronic illnesses, individuals with mental illnesses, migrants, refugees, and sex workers. At the beginning of the pandemic, older people were focused upon in terms of protecting them from the virus. In a United Kingdom article, intersecting identities in older people in terms of non-White ethnic/racial groups, disabilities, chronic illnesses, non-heterosexual orientation, or living arrangements, such as residential care, were stressed, as they were hardly considered in policies and mitigation measures (Buffel et al., 2021). These intersections may exacerbate adversities for older people and increase vulnerability in terms of loss of connection, support, and increase in isolation and loneliness. This may be particularly pronounced in deprived neighborhoods, as discussed in the United Kingdom (Buffel et al., 2021). Individuals with chronic mental illness also have to be considered with regard to their intersecting identities. Some identities might be connected to an increased risk of vulnerability: gender and sexual identity minorities, BIPOC, refugees or having a migration background, and individuals with lower income or living in poverty reported worse mental health in China, the United States, and the United Kingdom (Bhaskar et al., 2020; Gibson et al., 2021). Particularly, restricted access to (non-emergency) healthcare and isolation increased experiencing threats during the pandemic in psychiatric patients in the United Kingdom and the United States (Diaz et al., 2021; Leeming et al., 2022). Similarly, in the United States, gender-diverse individuals experienced more distress and less resilience (Salerno et al., 2020; Hunt et al., 2021), and more social isolation and interpersonal problems in terms of not being able to live their authentic self (Mitchell et al., 2022). Also, transgender women experienced increased inequality and hardship because of homelessness, unsafe jobs, and thus less possibility to protect themselves from the virus in the form of physical distancing. The effect of the pandemic and its mitigating measures further increased vulnerability in terms of, e.g., increasing poverty in the United States (Poteat et al., 2020). Employment was also an issue for migrant workers who might have had precarious working arrangements or were not documented; thus they experienced increased vulnerability because of the unsafe status of immigration in Canada, Germany, Latin America, India, South Korea, Nepal, and Nigeria (Lam, 2020; Chackalackal et al., 2021; Lotta and Kuhlmann, 2021). Overall, a lack of resources increased the vulnerability of diverse and marginalized groups in Malaysia and the United Kingdom (Bhaskar et al., 2020; Aung et al., 2021) but also, measures to contain the virus intensified the vulnerability to adverse outcomes of the pandemic. To avoid further discrimination and increase in vulnerability, any measure needs to be critically examined regarding potential misuse and abuse in terms of, e.g., profiling and further marginalization of individuals and social groups (Bhaskar et al., 2020).

### Resilience

**FIGURE 3:**
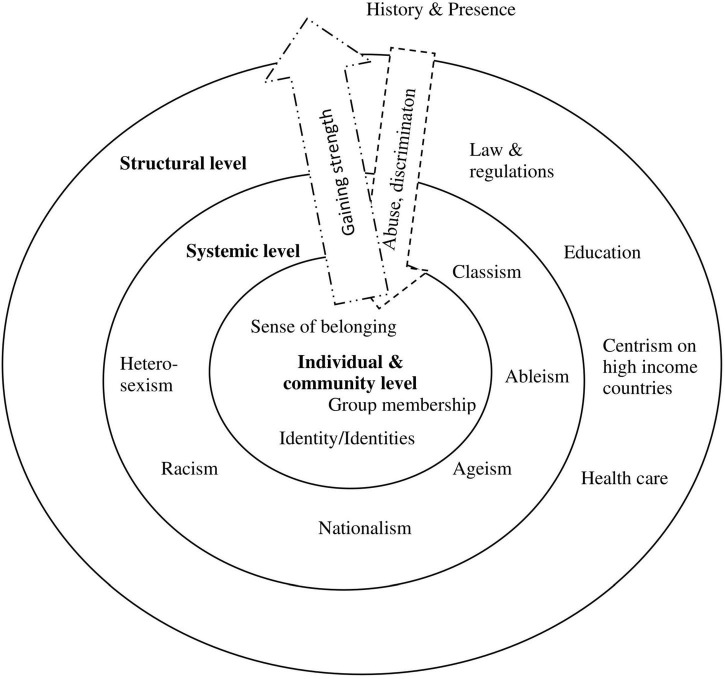
Resilience across levels.

Resilience is often seen as a possibility to overcome vulnerability. During the pandemic, resilience is sought after to ease adverse outcomes for individuals (Goldbach et al., 2021). Resilience in the context of social inequality was mostly understood as a social mechanism, where the individual and systemic levels interact; however, it also included chrono-levels in terms of resources and strength drawn from the history of communities (refer to Figure 3).

#### Structural Level: Politics and Education

Relying on grassroots organizations and individual commitments to provide support and connectedness to individuals of a community was not seen as sufficient from a Latin American perspective (Andia and Chorev, 2021). An approach at a structural level was needed, such as political engagement, to recognize and integrate such grassroot and individual responses in an overall strategy to support communities, as discussed in Malaysia and Canada (Daud, 2021; Morgan et al., 2022). Structural levels also comprised long-term strategies in terms of adjusting educational materials to include diverse social groups. This referred to promoting social resilience in terms of providing information and education to various social groups beyond majority and dominant social groups (Mahon and Mahon, 2021). Nevertheless, structural levels were also influenced by individual commitments. In this sense, online meetings and online communities potentially increased the visibility of marginalized groups and influenced policies and structural vulnerability, as shown in a Nigerian article (Oginni et al., 2021). Online presence was connected to less risk and exposure because of not meeting face-to-face. Additionally, online meetings had a wider range for meeting with others and increasing the visibility of adversities or harm (Oginni et al., 2021). Thus, individual and community engagements were impacting structures and reduced vulnerability at a structural level.

#### Systemic Level: Overcoming Classism and Racism With Community Resources

Children, particularly in low-income and poor environments, were especially vulnerable to the (social) impact of the pandemic. Loss of education and social interaction, safety, and care were seen as central aspects of the impact. Thus, hardship and disruption due (but not limited) to COVID-19 affected those with low income and ethnic or racial minority groups more often. Schools or other formal educational systems provided children with positive and nurturing relationships, which Herbers et al. (2021) also referred to as adaptive systems. Due to restrictions in accessing these systems, particularly at the onset of the pandemic, children in less nurturing environments may have been exposed to even more disruptions. For example, connection and resources could be provided by or in schools. In this sense, schools could and should be hubs for exchanging resources and connect, as demonstrated in a United States article (Sullivan et al., 2021), thus moving beyond a mere institution to educate.

To promote connection and build on the efficacy of communities, central figures provided support and connectedness as well as information on and guidance in overcoming the pandemic. Such central figures were related to religious leaders in Israel (Slobodin and Cohen, 2020), local leaders, and representatives of communities in Canada (Waruszynski et al., 2021). Thus, any measure to contain the spread of COVID-19 should be coordinated with religious needs and in cooperation with religious leaders. In Israel, it was noticed that some initial measures stood in contrast to religious rules, but later measures included cooperation with religious groups to contain the spreading of the virus (Slobodin and Cohen, 2020). Thus, religious leaders supported the measures by calling for, e.g., prayers in families instead of larger groups. Other important figures in Canada were local leaders and representatives of diverse communities to reduce further marginalization and to empower communities by providing them with collective efficacy (Waruszynski et al., 2021). Barriers to participation and collective and self-efficacy of communities and individuals were not only present at individual levels but also related to systemic challenges. In this sense, understanding systemic constraints was important to implement interventions and increase resilience and mitigate adversity as discussed in the United States (Blustein et al., 2021).

Regarding low-income parents or ethnic or racial minority groups, it was found that physical distancing measures or quarantines were interwoven with privilege of space or resources. Privilege in terms of higher income and higher socioeconomic status protected individuals from adverse impacts of the pandemic in China (Wu et al., 2021). Higher socioeconomic status, hence higher class, granted access to hygiene supplies, consisted of financial security due to non-precarious employment status, and provided access to a community that offered further resources.

#### Community and Individual Levels: Strength From the Community and (Historical) Hardship

In LGBTQI2S+ (lesbian, gay, bisexual, transgender, queer/questioning, intersex, two spirited and other not named sexual and gender identities) populations, participants referred to resilience because of lessons learned from previous experiences with hardship, e.g., in the United States (Abreu et al., 2021; Gonzalez et al., 2021; Quinn et al., 2021), and radical acceptance of, e.g., their identity, and the everyday commitment to one’s identity and acknowledgment of having a privileged position with access to resources and care (Gonzalez et al., 2021). Resilience referred to embracing collective and individual aspects at such a meta-level. Cheng et al. focused on their tripartite collective psychosocial resilience model in collective efforts to be supported as well as to differentiate between personal–individual and collective aspects. The three arms of their model include critical consciousness of discrimination as common fate, critical consciousness-informed racial/ethnic identity, and advocacy (Cheng et al., 2021). According to their explications, perceiving and realizing racism as collective or common fate instead of targeting a specific individual, as well as committing or embracing one’s ethnic identity and advocacy and solidarity in the community or group, may buffer the impact of (COVID-19 related) racism. Similarly, in a Canadian study, younger LGBTQI2S+ individuals also experienced support in online communities and strength from sharing information and educating others about, e.g., pride (Hiebert and Kortes-Miller, 2021). They also contributed to building and providing social resources by caring for the community. In turn, this also meant that, in an example from the United States, they benefited from giving, which increased their own feeling of resilience (Gonzalez et al., 2021) and collective and self-efficacy.

Sex workers and, particularly, migrant sex workers were additionally and intersectionally affected by discrimination, racism, and immigration status, which shaped their precarious situation and vulnerability. However, sex worker communities have proven to be resourceful and to rapidly respond to supporting sex workers in Canada (Lam, 2020). Thus, support by the community had various effects: it strengthened individuals in the community by providing them with resources, and it increased self-efficacy in individuals offering support to others. Resilience was experienced as a person-by-context interaction from a United States perspective (Herbers et al., 2021), thus highlighting that the context and the environment are detrimental in an individual’s display or activation of resilience. A United States study also showed that optimism and resilience as traits were beneficial, as these characteristics buffered the impact of loss of resources on mental health in the elderly and chronically ill people (McElroy-Heltzel et al., 2022).

## Discussion

In this article, we searched for resilience and vulnerability in minority and marginalized groups during the COVID-19 pandemic. The pandemic may be viewed as a crisis or as trauma to grasp its impact. Sullivan et al. even referred to it as a disaster (Sullivan et al., 2021), which is also accurate as a disaster is conceptualized as an interplay between the hazard as such and its social impact (Wisner et al., 2004). Social consequences and increasing social divide in societies resulting from the pandemic were also mentioned at the onset of the pandemic (World Health Organization [WHO], 2020b) and are a global concern.

In our review, we found that the impact of the pandemic on social inequality is referred to in terms of the impact of mitigating measures to contain the spread of the virus or the government neglecting to implement timely measures. Derived from the analysis of the literature, it is the context of mitigating measures, which exacerbate social inequality and potential vulnerability [cf (Cutter et al., 2008)]. We argue that it is *potential* vulnerability, as we see (1) vulnerability in interaction with resilience but not in opposition to resilience and (2) vulnerability through structures and systems as having the potential to increase adverse effects on individuals and communities. In this sense, we do not see vulnerability as an irrevocable outcome for minority and marginalized groups but as a product of structures and systems creating adversity for minority and marginalized groups and individuals.

Based on our review, we deduced three working hypotheses:

(1) Social inequality must be considered at a global level: inequality at a global level translates into a vulnerable context for an individual.

Whereas one focus has been on the impact of the pandemic on social inequality and roots of social inequality in systems of oppression, studies have also shown that we need to address inequality at a global level. For example, the unequal distribution of jobs that may be performed at home is not only evident at a national level but also in comparison between higher- and lower-income countries (Sanchez et al., 2021). Similarly, in their review on six countries ranging from Mexico and Colombia to Nigeria, South Korea, India, and Nepal, Chackalackal et al. (2021) concluded that measures to contain the virus focused on possibilities in high-income countries but were not necessarily translatable to lower-income countries. Additionally, the effects of climate change may affect those in crowded housing and with less access to urban green spaces and increase inequality even further, as discussed in an article with United States focus (Bikomeye et al., 2021). Urban green spaces are enjoyed in varying degrees in different countries, as urban green spaces may be restricted in their access (García de Jalón et al., 2020) or larger, desirable green spaces may not be available (Wendel et al., 2011). Therefore, social inequality in terms of privilege and oppression should not only be considered at societal and individual levels and with regard to the specific context of a country or a state. Inequality should be additionally assessed across countries and continents, thus on a global scale. Social inequality at a global level appears to be a larger representation of structural and systemic levels creating social inequality and vulnerability at societal and individual levels, and vice versa. This also implies that studies should provide more contextualized information, so future studies building on the findings can further investigate potential global and potential context/country-specific aspects. Thus, we need to consider the global impact on national inequality.

(2) Vulnerability is historically situated: vulnerability (experienced in the pandemic) is maintained and reinforced by history.

The COVID-19 pandemic is not just a crisis at an international level impacting marginalized and minority groups to a great extent. It may also be experienced in relation to collective and historical trauma. Such collective trauma is experienced differently by diverse groups across countries (e.g., Canada, United States, and Arabic countries) and exacerbates the feelings of vulnerability or threat (Diaz et al., 2021; Kira et al., 2021; Waruszynski et al., 2021). Additional stressors, such as murders of members of the Black and people of color community, further impede a sense of safety and contribute to the history and transgenerational trauma in the community, as shown by an example from the United States (Herbers et al., 2021). Vulnerability must be understood with its historic roots that are noticeable in today’s social inequality. Thereby, potential vulnerability is transported by means of education, regulations, and measures to contain COVID-19, housing, and employment, and working condition, which are built on majority and high-income, thus privileged, context and neglect minority and marginalized voices (Mahon and Mahon, 2021). Whereas this potential vulnerability is situated at structural levels, it is translated to social groups and individuals through systems of oppression, such as racism, classism, ableism, and heteronormativity. The implications of what is defined as vulnerable and vulnerability has direct relevance for diverse social groups and individuals (Hutcheon and Lashewicz, 2014). By perpetuating the history of abuse and discrimination of marginalized groups, inequality is reinforced (Krishnan et al., 2020) and not reduced [refer also to Pain (2020)]. These mechanisms are noticeable in and maintained at the communal, societal, national, and global levels. Intensifying interdisciplinary approaches, e.g., Black, postcolonial, queer, and feminist theories and perspectives, in (e.g., health) research will deepen the knowledge of the meaning of vulnerability and further evolve concepts and mitigation strategies to decrease inequality during a pandemic or other crisis.

(3) Strength through collective (historical) hardship: vulnerability is not the opposite of resilience but may serve as an aspect of resilience.

Vulnerability is not (only) seen in terms of hardship but also in terms of empowerment. For example, Gao and Sai (2021) stated, that “[w]riting through vulnerability liberates us to heal, to calm down, and to find meanings in our lived experiences” (p.188). In the United States, marginalized and minority groups found strength and resilience in their previous experiences with hardship (Gonzalez et al., 2021; Quinn et al., 2021), acknowledgment of collective fate (Cheng et al., 2021), and the history of their community or their ancestors (Abreu et al., 2021). Similar to revisiting the history of discrimination and abuse of marginalized groups, acknowledging these as well as contextualizing current vulnerability and inequality from such a historically informed perspective (Krishnan et al., 2020), resilience must be understood in terms of historically created vulnerability and through such vulnerability. Renewed or newly found connection to one’s community (Mahon and Mahon, 2021), identification with values of a community (Hiebert and Kortes-Miller, 2021), and advocacy for (other) marginalized groups benefit (Abreu et al., 2021; Gonzalez et al., 2021; Quinn et al., 2021) resilience. This underscores the importance of feeling part of a community and a sense of belonging to a community. Resilience and interventions to stimulate or increase resilience referred to advocacy, connectedness, social support, and collective action to stimulate efficacy. This emphasized the interaction between an individual and the environment (Herbers et al., 2021). Self- and collective efficacy and social connectedness are well-established aspects fostering resilience in disasters (Hobfoll et al., 2007) and are prevalent in studies reviewed in this context. Beyond these aspects, identity and historical embedding of vulnerability and inequality may be particularly important for minority and marginalized and groups and individuals [refer to, e.g., (Hutcheon and Lashewicz, 2014)]. Thus, resilience has been found in the context of previous experiences with vulnerability (Gonzalez et al., 2021). This is particularly true if referring to vulnerability as discussed by Baiasu (2020), who also saw vulnerability as openness. Such openness may be discussed in terms of having experienced vulnerability, which in turn did not result in personally closing up to protect oneself but embracing these experiences to build strength. Further endeavors should acknowledge Black, postcolonial, indigenous, queer, and feminist perspectives in trauma and resilience research, as they have already pointed out the role of collective trauma and its social, political, and structural implications (Pain, 2020, 2021) but are not well-integrated in many trauma and resilience research studies. Additionally, it shows that multiplicity of resilience and resilience narratives has to be acknowledged and understood in their contextual, subjective, and constructivist embedding (Hutcheon and Lashewicz, 2014; Powell et al., 2014).

### Fundamental Issues as Way Forward

This review underscores the necessity to acknowledge historical roots, definitions, and scope of fundamental concepts. As shown above, Hobfoll et al. (2007) showed distinct elements of resilience in disasters and large-scale emergencies. Some of these aspects can also be traced in this review. However, there appear to be distinct features of resilience in minority and marginalized groups, such as the role and acknowledgment of history and historical discrimination (Pain, 2020), having confidence in one’s identity and standing by one’s identity/identities, and finding strength in past adversities from ancestors and communities. Focusing on vulnerability and resilience has shown that these are not distinct entities, but that they are interconnected. Vulnerability is, thus, not a secluded entity and in opposition to resilience, which marks the desirable state. Whereas resilience is, per definition, embedded in the context of adversity and, thus, potential vulnerability (Werner, 2005; Rutter, 2012; Juen and Siller, 2013; Luthar et al., 2015; Baiasu, 2020; Kubacki et al., 2020), vulnerability is hardly understood as an integral part of resilience (Baiasu, 2020). Thus, future endeavors should focus on the meaning of vulnerability at the individual, systemic, and structural levels, and its implications for resilience. Intersectionality [e.g., (Moradi and Grzanka, 2017)] and socioecological models [e.g., (Bronfenbrenner, 1977; Bronfenbrenner and Evans, 2000)] provide useful frameworks to consider levels and interconnections in research. Such frameworks, even if not all levels are applied in a study, may elicit the specificity of findings and outcomes [refer to, e.g., (McCollum et al., 2019)]. The gained knowledge may help to embed and contextualize experiences at the individual and group-based levels regarding structural and historical elements and vice versa.

This review is not without limitations. The rapidly increasing number of articles on this topic may have led to missing important contributions to this field of research. However, even though we are confident that we have captured the most important aspects of vulnerability and resilience in the social inequality context, we cannot rule out that we missed recent developments. Additionally, we used English search terms, which marginalizes publications published in other languages or published in other databases. This reinforces discourses in academia in which specific databases, languages, and institutions are dominant at the expense of others. As was also noticeable in the selected literature, one dominant discourse focuses on the United States. In reflection of social inequality during the COVID-19 pandemic, the increasing publication rate might also increase further social disparities in knowledge production. Many articles focused on minority or marginalized groups, as did we. However, this also supports an underlying (unnamed) assumption of having a reference group, which possesses power, resilience, and privilege. Critiques on the wording of minority and majority have been articulated by others as well (Seyranian et al., 2008). It is, thus, important to continuously take up a critical position toward terminology. Another limitation refers to the authors as researchers in the discussion of social inequality. Research findings also relate to the authors as researchers, our professional socialization, and epistemological understanding. By reflecting on these aspects in the authors as researchers, we are likely to advance our understanding of concepts of vulnerability and resilience in minority and marginalized groups across countries and locations. Also, by doing so, we might be more aware of how and when we reproduce social inequality or silence voices. Thus, the underlying perspective in this review also refers to speaking from a place of privilege as we (HS and NA) are, in many references, part of the “dominant” group, e.g., through our position as researchers.

## Conclusion

Our review highlights that we need to critically review which voices have been neglected in the development of concepts. Gaillard and Mercer (2012) emphasized that “our inability to bring all actors, usually working at different scales and in dissimilar directions, together around the same table” (p. 101) causes a lack of collaboration between different actors in creating a dialog (Gaillard and Mercer, 2012). This statement bears resemblance to our review: diverse voices need to be visible in models and concepts focusing on marginalized and minority groups, vulnerability and resilience in the COVID-19 pandemic (as one example for adversities). As shown, resilience includes vulnerability in terms of acknowledging discrimination as collective and common fate (Cheng et al., 2021), in terms of historical discrimination, and in terms of structures at a global level that are reflected in each society and impact individuals. Understanding collective trauma in marginalized groups and its contribution in increasing vulnerability and inequality (Cheng et al., 2021; Kira et al., 2021) is important; another side of collective trauma from a present and historical point of view refers to providing strength and confidence (Baiasu, 2020; Abreu et al., 2021; Gonzalez et al., 2021; Quinn et al., 2021). Critical acceptance of one’s identity (Gonzalez et al., 2021) or identities, as well as standing by these may provide a sense of belonging to the community. In relation to collective and historical trauma, such identities and belonging might elicit specific and unique constructions of resilience in minority and marginalized groups and individuals, as is also proposed by feminist, Black, queer, and indigenous approaches [refer to Burstow (2003); Tseris (2013); Pain (2020)].

## Author Contributions

HS and NA have both substantially contributed to writing this article, critically reviewed the findings, and discussed and revised the draft. HS conceptualized the idea, performed the search, and wrote the first draft of the manuscript. Both authors contributed to manuscript revision, read, and approved the submitted version.

## Conflict of Interest

The authors declare that the research was conducted in the absence of any commercial or financial relationships that could be construed as a potential conflict of interest.

## Publisher’s Note

All claims expressed in this article are solely those of the authors and do not necessarily represent those of their affiliated organizations, or those of the publisher, the editors and the reviewers. Any product that may be evaluated in this article, or claim that may be made by its manufacturer, is not guaranteed or endorsed by the publisher.
